# Energy Transfer-Based Recognition of Membrane Cholesterol by Controlling Intradistance of Linker

**DOI:** 10.3390/s24072315

**Published:** 2024-04-05

**Authors:** Yong Ho Cho, Tae Kyung Won, Dong June Ahn

**Affiliations:** 1 Department of Chemical and Biological Engineering, Korea University, Seoul 02841, Republic of Korea; cyh9312@korea.ac.kr (Y.H.C.); callys@korea.ac.kr (T.K.W.); 2 KU-KIST Graduate School of Converging Science and Technology, Korea University, Seoul 02841, Republic of Korea

**Keywords:** cyclodextrin, host-guest interaction, gold nanoparticle quenching, surface modification, turn-on sensing

## Abstract

Gold nanoparticles (AuNPs) are good candidates for donor material in energy transfer systems and can easily be functionalized with various ligands on the surface with Au–S bonding. Cyclodextrin (CD) forms inclusion complexes with fluorophores due to its unique structure for host–guest interaction. In this study, we fabricated βCD-functionalized AuNPs using different lengths of thiol ligands and recognized cholesterol to confirm the energy-transfer-based turn-on fluorescence mechanism. AuNP–βCD conjugated with various thiol ligands and quenched the fluorescein (Fl) dye, forming βCD-Fl inclusion complexes. As the distance between AuNPs and βCD decreased, the quenching efficiency became higher. The quenched fluorescence was recovered when the cholesterol replaced the Fl because of the stronger binding affinity of the cholesterol with βCD. The efficiency of cholesterol recognition was also affected by the energy transfer effect because the shorter βCD ligand had a higher fluorescence recovery. Furthermore, we fabricated a liposome with cholesterol embedded in the lipid bilayer membrane to mimic the cholesterol coexisting with lipids in human serum. These cellular cholesterols accelerated the replacement of the Fl molecules, resulting in a fluorescence recovery higher than that of pure lipid. These discoveries are expected to give guidance towards cholesterol sensors or energy-transfer-based biosensors using AuNPs.

## 1. Introduction

Gold nanoparticles (AuNPs) have been widely used in bioapplications because of their useful properties, such as size- and shape-dependent optoelectronics [1,2], simple and multi-functionalization [3], plasmonic effect [4], good biocompatibility [5,6], and low toxicity [7]. The surface chemistry of AuNPs is crucial in functionalizing various ligands on their surface [8,9,10,11]. To use the specific Au–S bond with a thiolated ligand or thiol functional group, AuNPs can be functionalized with various bioligands, such as DNA [12,13], peptide [14], antibody [15], antigen [16], lipid [17], and enzyme [18]. Additionally, AuNPs are good candidates as donor materials due to their good quenching efficiency in a wide range of wavelengths in the visible light area for energy transfer [19,20,21]. According to Föster’s theory, the rate of energy transfer is considerably affected by the distance between the donor and acceptor [22,23,24,25].

Cyclodextrin (CD) is an oligosaccharide consisting of a cyclic ring structure of glucose subunits. It has three different sizes of ring structures depending on the number of glucoses: six for α-cyclodextrin (αCD), seven for β-cyclodextrin (βCD), and eight for γ-cyclodextrin (γCD). It can form inclusion complexes with various molecules, known as host–gust interaction, due to its unique structure with a hydrophobic cavity and hydrophilic outside [26]. Depending on the type of CD or modification of functional groups, binding affinity with guest molecules is different [27,28,29]. When multiple guest molecules exist, various inclusion complexes are formed depending on their biding affinity, or the original guest material is replaced with a new guest material [30,31,32].

**Scheme 1:**
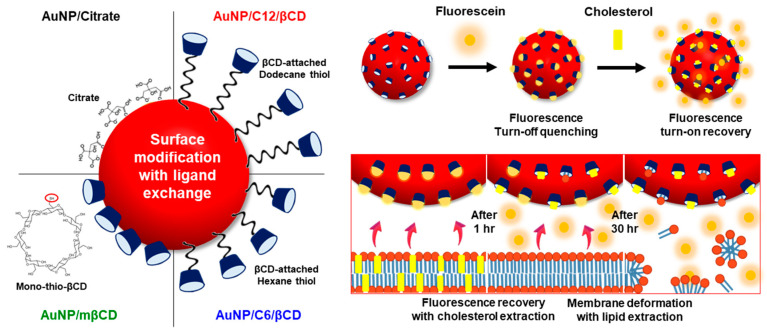
Schematic illustrations of turn-on recognition of monomeric and membrane cholesterol with β-CD-attached AuNPs with different intradistance of linker.

In this research, we fabricated βCD-functionalized AuNPs and formed βCD-fluorophore inclusions with the fluorescein (Fl) dye molecule. Various thiol functionalized ligands were used to confirm the energy transfer efficiency according to the distance between βCD and AuNP [30]. The other guest molecule as the target material was cholesterol, an important component of animal cell membranes and the main precursor for the synthesis of biomolecules, having great binding affinity with βCD. For more details, schematic illustrations of turn-on recognition of cholesterol with βCD-attached AuNPs are shown in Scheme 1. After synthesizing spherical gold particles with a size of 20 nm, βCD was attached to the surface of the AuNPs through a thiol-ligand reaction. Three thiol ligands of different lengths were used for the surface modification of gold particles. We used dodecane thiol (12MPA), hexane thiol (6HPA), and mono-(6-mercapto-6-deoxy)-beta-cyclodextrin (mβCD) to adjust the distance from the AuNPs. When fluorescein (Fl) was added to the AuNP solution, the fluorescein was incorporated into the βCD to form a βCD-Fl inclusion complex. The fluorescein molecules that were close to the AuNPs underwent fluorescence quenching through energy transfer to the AuNPs. After cholesterol (Chol), which has a higher binding affinity with βCD than fluorescein [33,34], was added, it replaced fluorescein and constituted a βCD-Chol inclusion complex. The replaced fluorescein molecule moved away from the AuNPs, and the recovered fluorescence created a turn-on recognition signal. We fabricated cholesterol-mixed liposome nanoparticles as a membrane cholesterol model to mimic the environment in which cholesterol coexisted with lipids in human epithelial cells [35]. The liposome nanoparticles interacted with the three types of AuNPs-βCD, including Fl, for the recognition of cellular cholesterol. The cholesterol could interact with βCD faster than lipids, leading to a higher recovered fluorescence intensity according to the reaction time. When liposomes were exposed to βCD for a long time, the structure of the liposomes collapsed due to the interaction of lipid and βCD. Through cholesterol detection with βCD-functionalized AuNPs, we confirmed that the distance between the AuNPs and fluorophores affects energy transfer and detection efficiency.

## 2. Materials and Methods

### 2.1. Preparation of 20 nm Spherical AuNPs

Gold(III) chloride trihydrate (≥99.9%, trace metals basis, Sigma-Aldrich, St. Louis, MO, USA) with a concentration of 1 mM was dissolved in deionized water (20 mL). The solution was heated at 100 °C with 600 rpm stirring. Sodium citrate tribasic dehydrate (ACS reagent, ≥99%, Sigma-Aldrich) with a concentration of 38.8 mM was dissolved in deionized water (2 mL) and added to the boiled gold solution quickly. After approximately 10 min, the solution turned dark red and was cooled to room temperature. Then, the solution was filtered using a syringe filter (0.22 µm pore, MCE membrane, Biofil, Indore, India) and stored at 4 °C before use.

### 2.2. β-Cyclodextrin Modification of AuNPs with Thiol Ligands

To control the distance between β-cyclodextrin and the gold surface, we used 4 thiol ligands: 12-mercaptododecanoic acid (MPA, >96%, Sigma-Aldrich), 6-mercaptohexanoic acid (MHA, >90%, Sigma-Aldrich), heptakis-(6-mercapto-6-deoxy)-β-cyclodextrin (hSH-β-CD, >98%, Zhiyuan Biotechnology, Binzhou, China), and mono-(6-mercapto6-deoxy)-β-cyclodextrin (mSH-β-CD, >98%, Zhiyuan Biotechnology). For MPA, 50 µL of MPA was added to 1 mL of AuNP solution. After reacting for 3 h, the solution was centrifuged at 12,000 rpm for 10 min and dispersed in deionized water. Centrifugation was repeated twice. Subsequently, 10 µL of 5 mM 1-ethyl-3-(3′-dimethylaminopropyl) carbodiimide · HCl (EDC, Thermo Scientific), 10 µL of 5 mM N-hydroxysuccinimide (NHS, 98%, Sigma-Aldrich), and 50 µL of 1 mM 3A-amino-3A-deoxy-β-cyclodextrin hydrate (NH_2_-β-CD, >97%, Tokyo Chemical Industry, Portland, OR, USA) in deionized water were added to the solution in order. After reacting for 3 h, the solution was centrifuged at 12,000 rpm for 10 min and dispersed in deionized water. Centrifugation was repeated twice, and the solution was stored at 4 °C before use. For HPA, the process was the same as that for MPA.

In the case of mSH-β-CD, 50 µL of mSH-β-CD was added to 1 mL of AuNP solution. After reacting for 3 h, the solution was centrifuged at 12,000 rpm for 10 min and dispersed in deionized water. Centrifugation was repeated twice, followed by storage at 4 °C before use. For hSH-β-CD, the process was the same as that for mSH-β-CD.

### 2.3. Fluorescence Turn-Off Quenching by Inclusion of Dye with β-Cyclodextrin

Fluorescein (Sigma-Aldrich) with a concentration of 1 mM was added to deionized water (20 mL) and sonicated until completely dissolved. The fluorescein solution was diluted to 100 µM with deionized water. Subsequently, 50 µL of fluorescein solution was mixed with 950 µL of β-cyclodextrin-modified AuNP solution for 2 h. The optical responses of the samples were analyzed.

### 2.4. Preparation of Cholesterol Embedded Liposome Particles

The fabrication of artificial liposome particles was referred to by previous work [36]. [1,2-dioleoyl-sn-glycero-3-phosphocholine (DOPC, >99%, Avanti Polar Lipids, Alabaster, AL, USA), 1,2-dipalmitoyl-sn-glycero-3-phosphocholine (DPPC, >99%, Avanti Polar Lipids), and cholesterol (≥99%, Sigma-Aldrich) were dissolved in chloroform (2 mL). The solution was filtered using a syringe filter (0.2 µm pore, PTFE membrane, Macherey-Nagel, Dueren, Germany) and dried using N_2_ gas. Deionized water (10 mL) was added to the dried sample and heated in an 80 °C bath for 15 min. Then, the solution was sonicated at 10 mW (Sonic Dismembrator 500, Fisher Scientific, Hampton, USA) for 15 min and stored at 4 °C before use. The unstructured lipids and cholesterols were washed by centrifugation at 12,000 rpm for 10 min.

### 2.5. Cholesterol Detection with Fluorescence Turn-On Recovery

The turn-on fluorescence recovery experiment was referred to by previous work [37,38]. Cholesterol with a concentration of 10 mM was dissolved in ethanol (1 mL). The solution was diluted to 1 mM with deionized water. Next, 50 µL of cholesterol solution (0.1 v% ethanol) was mixed with 950 µL of quenched gold solution. Subsequently, 50 µL of 1 mM liposome solution was also mixed with 950 µL of quenched gold solution. After reacting for 2 h, the optical responses of the samples were analyzed.

### 2.6. Characterization

The surface morphology of the AuNPs was observed using transmission electron microscopy (TEM, Technai G2, FEI, Jülich, Germany). The surface morphology of the lipid vesicles was observed using scanning electron microscopy (SEM, S-4300, Hitachi, Tokyo, Japan). The optical properties were measured by UV-Vis spectroscopy (Cary 60 UV-Vis, Agilent Technology, Santa Clara, CA, USA) and fluorescence spectroscopy (G9800A, Agilent Technology). The surface charge and hydrodynamic diameter (HDD) were analyzed using electrophoretic light-scattering spectroscopy (ELS-Z2, Otsuka Electronics, Osaka, Japan).

## 3. Results

### 3.1. Fabrication of βCD-Modified AuNPs with Thiol Ligand Exchange

We fabricated approximately 20 nm-sized AuNPs by citrate and HAuCl_4_, commonly known as the Frens method [39]. Citrate served as a capping agent to reduce gold ions at high temperatures and as a stabilizer to lower the surface energy of the AuNPs. Citrate could be replaced with thiol ligand through Au–S bonding, a strong chemical bond that replaced the ionic bond of Au–citrate. We used 12-mercaptododecanoic acid (MPA), 6-mercaptohexanoic acid (MHA), and mono-(6-mercapto6-deoxy)-β-cyclodextrin (mβCD) as a thiol ligand. In the case of MPA and MHA, we conjugated the βCD to the AuNP surface through a terminal carboxyl group capable of an EDC/NHS coupling reaction with amine-βCD. Step-by-step absorption spectra and TEM images are shown in Figure S1. In the case of mβCD, the hydroxyl group of βCD can be chemically substituted with a thiol to be directly attached to the gold surface. Finally, we fabricated three types of AuNPs-βCD: AuNP/C12/βCD, AuNP/C6/βCD, and AuNP/mβCD.

**Figure 1:**
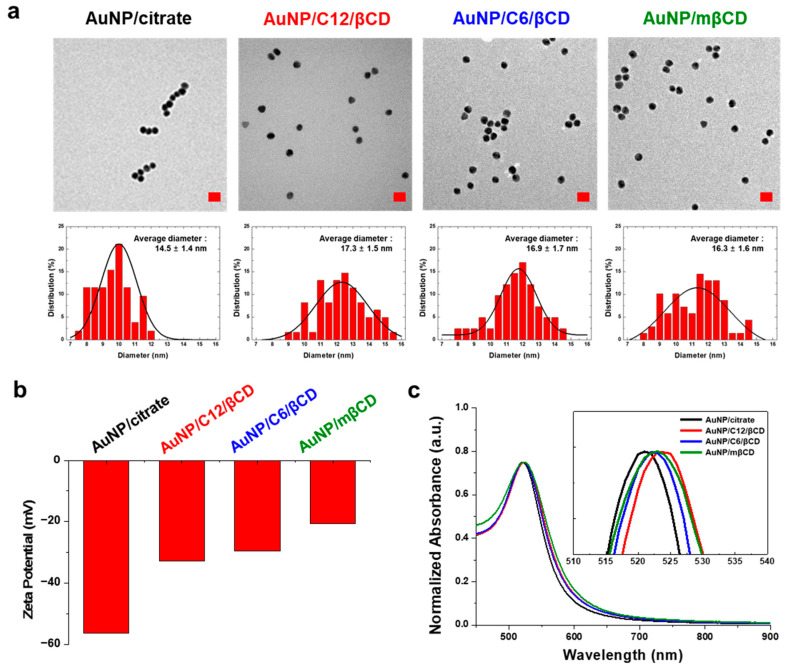
Surface modifications of AuNPs with βCD-attached thiol ligands. (**a**) Transmission electron microscopy (TEM) images and size distribution of βCD-functionalized AuNPs (scale bars: 20 nm). (**b**) Surface charge of AuNPs with different ligands. (**c**) Visible spectra of βCD-functionalized AuNPs.

The morphological and optical properties of the AuNPs-βCD were measured. TEM images of AuNPs-βCD are shown in Figure 1a. In all cases, the particles had a spherical shape. For quantitative analysis, the size of the particles in the image was measured. The average diameter of AuNP/citrate was 14.5 ± 1.4 nm, which was the smallest value, while AuNPs-βCD increased slightly, but the deviation was small: 17.3 ± 1.5 nm for AuNP/C12/βCD, 16.9 ± 1.7 nm for AuNP/C6/βCD, and 16.3 ± 1.6 nm for AuNP/mβCD. Dynamic light-scattering measurement was also performed, and the hydrodynamic diameter (HDD) of AuNPs-βCD is shown in Figure S2. HDD is larger than the actual diameter of the particle because it is the diameter of a potential layer composed of ions surrounding the particle [40,41]. The HDD of AuNP/citrate was 22.9 ± 11.6 nm and increased with modification of thiol ligands: 93.0 ± 74.6 nm for AuNP/C12/βCD, 78.1 ± 66.6 nm for AuNP/C6/βCD, and 32.7 ± 19.3 nm for AuNP/mβCD. The increase in HDD was greater in the relatively long ligand. The AuNPs combined with carbon chains, such as AuNP/C12/βCD and AuNP/C6/βCD, had a significantly higher HDD than AuNP/mβCD directly attached to the surface. The surface charge of AuNPs-βCD is also shown in Figure 1b. The surface charge of AuNP/citrate was −56.5 mV because of the high charge density of the citrate molecules. Meanwhile, it was observed that AuNPs-βCD had a relatively lower negative-charge value with a low charge density of βCD: −32.9 mV for AuNP/C12/βCD, −29.6 mV for AuNP/C6/βCD, and −20.7 mV for AuNP/mβCD. UV-Vis absorbance spectra of AuNPs-βCD are shown in Figure 1c. AuNPs have their own absorption peaks according to size due to the localized surface plasmon resonance (LSPR) effect. The maximum peak of the main absorbance band of AuNP/citrate, with a size of 20 nm, was at approximately 520 nm. The AuNPs-βCD caused a few nm red-shift, indicating that the thiol ligands were substituted with citrate on the surface.

### 3.2. Fluorescence Turn-On Recovery of AuNP-βCD-Fluorescein Complexes for the Detection of Monomeric Cholesterol

**Figure 2:**
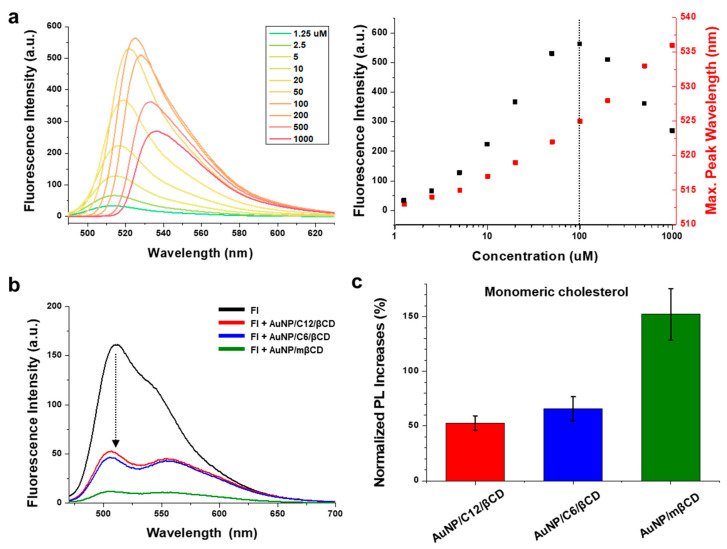
Fluorescence turn-off quenching and turn-on recovery with the Fl-βCD inclusion complex for monomeric cholesterol recognition. (**a**) Photoluminescence spectra of mixture of fluorescein dye (5 µM) and βCD-functionalized AuNPs. (**b**) Fluorescence turn-on recovery of βCD-functionalized AuNPs in response to monomeric cholesterol with replacement of inclusion molecules in βCD. (**c**) Calculated normalized PL increases (%) = (I − I_o_)/I_o_ × 100 (I: fluorescence intensity after cholesterol interaction at 512 nm; I_o_: fluorescence intensity before cholesterol interaction at 512 nm; concentration of cholesterol: 12.5 µM).

We chose fluorescein (Fl), a commonly known aggregation-caused quenching (ACQ) material, as a fluorescent donor because of its high binding affinity for βCD and approximately 520 nm fluorescence peak matched with the absorbance of the AuNP solution. The fluorescence spectra of various concentrations of Fl are shown in Figure 2a. From these results, under 100 μM of Fl was used when fluorescence decreased as the concentration decreased. The AuNPs-βCD was mixed with Fl dye to form a βCD-Fl inclusion complex. The fluorescence spectra of AuNPs-βCD interacted with Fl, as shown in Figure 2b. When Fl was combined with βCD, the AuNP quenched the fluorescence of Fl due to energy transfer. The calculated quenching efficiency was 67.4% for AuNP/C12/βCD, 71.3% for AuNP/C6/βCD, and 92.5% for AuNP/mβCD. This confirms that the closer the distance between βCD and AuNP, the higher the quenching efficiency.

Then, monomeric cholesterol dispersed in ethanol was added to the quenched solution. Monomeric cholesterol was complexed with βCD by replacing Fl due to its higher binding affinity with βCD than Fl. As Fl became distant from AuNPs, the fluorescence of Fl recovered. The recovered fluorescence spectra for monomeric cholesterol are shown in Figure S3, and the calculation of recovered fluorescence from the quenched state is shown in Figure 2c. The normalized Pl increases were 52.6% for AuNP/C12/βCD, 65.7% for AuNP/C6/βCD, and 152% for AuNP/mβCD. This showed that the higher the quenching efficiency, the greater the recovered fluorescence increase. The control case of the AuNP/citrate not attached to βCD is shown in Figure S4. The fluorescence intensity of Fl molecules was more quenched than other AuNPs-βCD due to the non-specific binding of Fl molecules on the surface of AuNP/citrate. However, the recovered fluorescence intensity was only 9% owing to the absence of specific binding domain with cholesterol, unlike the βCD molecule. As a result, if the distance between βCD and AuNP was closer, the quenching efficiency of fluorescence was improved, and the fluorescence increased more when it interacted with monomeric cholesterol.

### 3.3. Fabrication of Liposome Nanoparticles Containing Cholesterol as a Membrane Cholesterol Model

**Figure 3:**
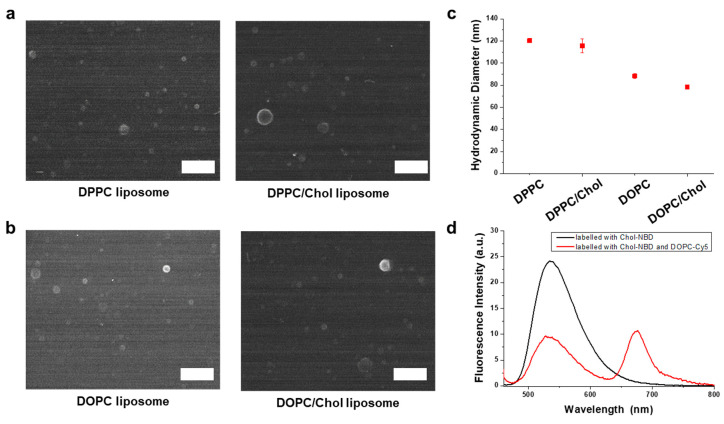
Fabrication of liposome particles with or without cholesterol. Scanning electron microscopy (SEM) images (scale bars, 1 µm) of 1 mM (**a**) DOPC, DOPC/Chol liposomes and (**b**) DPPC, DPPC/Chol liposomes. The molar ratio of lipid:chol with liposome was 3:1. (**c**) hydrodynamic diameter (HDD) of DOPC, DOPC/Chol, DPPC, and DPPC/Chol liposomes. (**d**) Photoluminescence spectra of DOPC/Chol liposomes labeled with NBD and Cy5 dyes.

We fabricated liposome nanoparticles consisting of phospholipid and cholesterol to make the environment similar to that of cholesterol in human epithelial cells. The artificial liposomes had the advantage of easily forming lipids and cholesterol uniformly in a self-assembled structure. We used dipalmitoyl phosphocholine (DPPC) and dioleoyl phosphocholine (DOPC) as phospholipids. As DOPC has a double bond in its carbon chain, it is more flexible and has a lower phase-transition temperature than DPPC, which has a single bond. The SEM images of liposome particles are shown in Figure 3a,b. All four types of liposomes had a spherical shape. There were some ruptured particles whose structure was destroyed by the dehydration force under the drying process, showing as bigger particles stuck to the substrate. The HDD of four artificial liposomes, cholesterol-free or 25% added, is shown in Figure 3c. While the diameter of the DPPC liposome was 120.3 nm, the DPPC/Chol liposome was smaller at 115.7 nm because cholesterol has a relatively smaller volume than lipids. The HDD was 88.3 nm for the DOPC liposome and 78.3 nm for the DOPC/Chol liposome. To determine the cholesterol distribution in liposome nanoparticles, we used NBD-labeled cholesterol (Chol-NBD) and Cy5-labeled DOPC (POPC-Cy5). The excitation and emission spectra of Chol-NBD and POPC-Cy5 are shown in Figure S5. If the emission of Chol-NBD and excitation of DOPC-Cy5 had an overlap region, Förster resonance energy transfer occurred when the distance between two molecules was less than 10 nm. The fluorescence spectra of DOPC/Chol liposomes labeled with dyes are shown in Figure 3d. Under excitation at 450 nm, the DOPC/Chol-NBD liposome emitted the fluorescence of NBD at 535 nm. In the case of the DOPC-Cy5/Chol-NBD liposome, the excitation energy was transferred from NBD to Cy5, emitting fluorescence at 535 and 675 nm.

### 3.4. Fluorescence Turn-On Recovery of AuNP-βCD-Fluorescein Complexes for Recognition of Membrane Cholesterol

**Figure 4:**
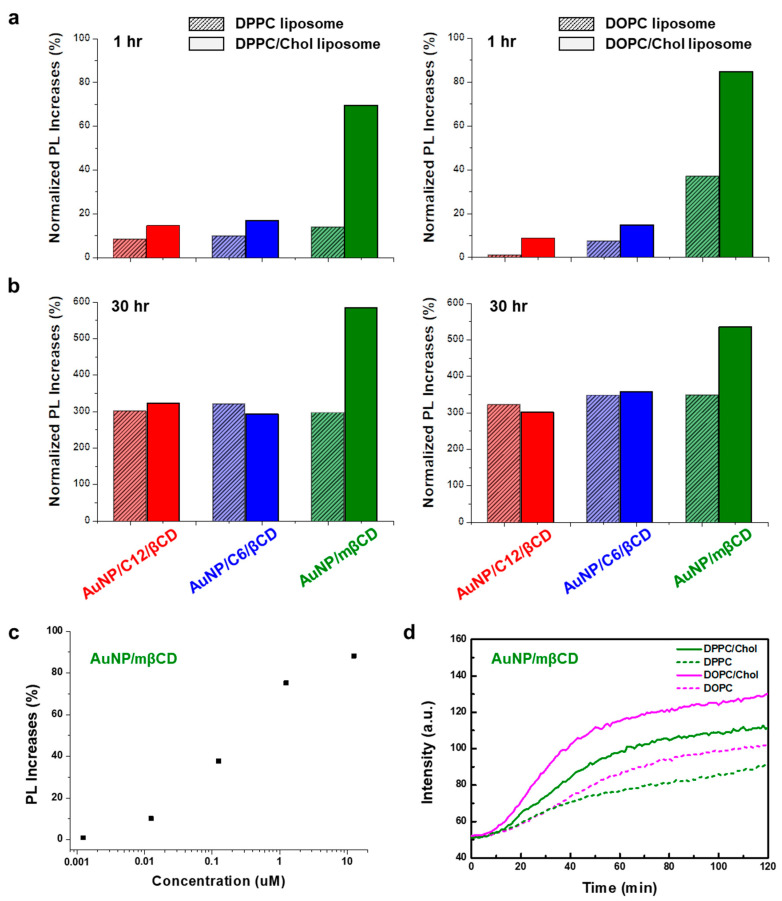
Cholesterol extraction in lipid membrane using βCD-functionalized AuNPs. Fluorescence turn-on recovery of β-CD functionalized AuNPs in response to cholesterol-embedded liposome particles after (**a**) 1 h and (**b**) 30 h. Normalized PL increases (%) = (I − I_o_/I_o_) × 100 (I: fluorescence intensity after cholesterol interaction at 512 nm; I_o_: fluorescence intensity before cholesterol interaction at 512 nm; concentration of cholesterol: 12.5 µM). (**c**) Photoluminescence intensities of AuNP/mβCD in response to DOPC/Chol liposomes with different concentrations of cholesterol. (**d**) Increase of photoluminescence of AuNP/mβCD in response to liposome particles over time.

Four types of liposome particles were treated with quenched AuNP-βCD-Fl complexes, and each spectrum of recovered fluorescence after 1, 2, and 30 h is shown in Figure S6. For quantitative analysis, the increase in recovered fluorescence was calculated and is shown in Table S1. The normalized PL increases after 1 h are shown in Figure 4a. In the case of DPPC liposome particles, the normalized PL increases were 8.4% for AuNP/C12/βCD, 9.8% for AuNP/C6/βCD, and 13.8% for AuNP/mβCD. This supports βCD interacting with DPPC lipid molecules, showing some recovered fluorescence. On the other hand, the normalized PL increases of DPPC/Chol liposome particles were 14.7% for AuNP/C12/βCD, 16.8% for AuNP/C6/βCD, and 69.6% for AuNP/mβCD, showing higher intensity than DPPC liposome in all cases. These results suggest that cholesterol was extracted by the βCD from the lipid membrane to make a βCD-Chol complex, further accelerating the exchange of the Fl molecules faster than lipid molecules. In particular, the AuNP/mβCD showed a substantial fluorescence increase of 69.6%. This means that AuNP/mβCD had a higher binding affinity with the DPPC/Chol liposome than other AuNPs/βCD particles. The case of interaction with DOPC liposome showed a similar trend to DPPC liposome, where cholesterol improves fluorescence recovery: 1.0% to 8.9% for AuNP/C12/βCD, 7.3% to 14.8% for AuNP/C6/βCD, and 36.9% to 84.7% for AuNP/mβCD. The normalized PL increases after 2 h are shown in Figure S7. The PL intensity was more recovered in all cases of the samples. However, the relative recovered rates with pure liposomes, such as DPPC and DOPC, were more increased than those with liposome with cholesterol, such as DPPC/Chol and DOPC/Chol. It was expected that the interaction between βCD and phospholipid was increasing while that with cholesterol was decreasing. The normalized PL increases after 30 h are shown in Figure 4b. This was enough time for all the lipids and cholesterol to react to βCD. The PL increases of liposomes with and without cholesterol were saturated at approximately 300%, except for AuNP/mβCD with liposomes. This means that all the Fl molecules in the βCD-Fl inclusion complex were escaped by phospholipids and cholesterol, resulting in the same fluorescence recovery with liposomes regardless of cholesterol. However, in the case of AuNP/mβCD, the PL increases were 296.6% for DPPC, 584.3% for DPPC/Chol, 348.0% for DOPC, and 535.9% for DOPC/Chol. The differences with these liposomes with or without cholesterol could be caused by steric hindrance of the lipid molecule interacting with AuNP/mβCD. The common inclusion complex of βCD-phospholipid has a structure in which the long carbon chain passes through the ring of two βCD molecules [28]. However, the distance was too short for DPPC and DOPC molecules to make an inclusion complex, with mβCD closely attached to the surface of AuNP. Therefore, the Fl molecules of the βCD-Fl inclusion complex could not escape fully in the case of pure liposomes like DOPC and DPPC, resulting in a difference from the liposomes with cholesterol. After treatment, we performed a nanoparticle tracking analysis (NTA). The scattered light of DPPC/Chol liposomes and 3D plot mapping of particle size are shown in Video S1 and Figure S8. In the case of the DPPC/Chol liposome before treatment, the scattered light showed that the size distribution of the liposomes was uniform, with a diameter of approximately 170 nm. After 1 h treatment with AuNP/mβCD, the size of most liposomes was maintained, but their uniformity was reduced. After 30 h treatment of AuNP/mβCD, most liposomes disappeared, and particles with size of few nanometers were observed. This could be due to the deformation of their bilayer structure by the interaction between lipid and βCD. To confirm the sensitivity of membrane cholesterol recognition, the AuNP/mβCD was treated with different concentrations of DOPC/Chol liposomes. The PL increase in samples is shown in Figure S9 and calculated in Figure 4c and Figure S10. The AuNP/mβCD was sensitive to changes in nanoscale concentration and could be detected from nano- to micro-molar concentrations of cholesterol. The kinetic PL analysis of AuNP/mβCD treated with liposomes is shown in Figure 4d. In all cases of liposomes, the fluorescence increased in the form of an unsaturated binding curve. The liposomes containing cholesterol increased fluorescence faster than pure lipid liposomes. As a result, we observed the fluorescence recovery of the AuNP-βCD-Fl complex interacted with liposomes being cholesterol-free or with 25% added. Cholesterol interacted with βCD faster than phospholipids to show higher fluorescence intensity, although the phospholipids became similar over time. There was no difference in fluorescence recovery according to the lipid, such as DOPC and DPPC.

## 4. Conclusions

We fabricated three types of AuNPs: AuNPs-βCD, AuNP/C12/βCD, and AuNP/C6/βCD with different distances between AuNP and βCD. We fabricated AuNPs conjugated with βCD and inclusion complexes with Fl molecules. The closer the distance between βCD and AuNP, the higher the energy transfer efficiency, showing a more quenched intensity of fluorescence. Similarly, after treatment of monomeric cholesterol, shorter ligands of AuNPs/βCD showed higher fluorescence recovery: 46% for AuNP/C12/βCD, 78% for AuNP/C6/βCD, and 128% for AuNP/mβCD. To determine the effect of membrane cholesterol, we fabricated artificial phospholipid liposomes that were cholesterol-free or 25% added and treated with the AuNPs-βCD-Fl complex. The βCD could also interact with DPPC lipid molecules, showing recovered fluorescence. Meanwhile, cholesterol accelerated the exchange of the Fl molecules, improving fluorescence recovery: 1.0% to 8.9% for AuNP/C12/βCD, 7.3% to 14.8% for AuNP/C6/βCD, and 36.9% to 84.7% for AuNP/mβCD in DOPC/chol liposome. In the case of the 30 h reaction, with enough time for all lipids and cholesterol to react with βCD, the PL intensity was recovered up to 348.0% for DOPC and 535.9% for DOPC/Chol, showing specific recognition of cholesterol. We expect this study to provide guidance for energy transfer-based sensors in biological applications.

## Data Availability

The data that support the findings of this study are available upon reasonable request from the authors.

## Supplementary Materials

The following supporting information can be downloaded at: https://www.mdpi.com/article/10.3390/s24072315/s1, Figure S1: Analysis of visible spectroscopy and transmittance electron microscopy (TEM) images in the process of AuNP/C12/βCD fabrication; Figure S2: Analysis of dynamic light scattering with AuNP-βCD particles; Figure S3: Photoluminescence spectra for recognition of monomeric cholesterol; Figure S4: Photoluminescence spectra of fluorescence quenching and turn-on recovery process with AuNP/Citrate; Figure S5: Optical properties of Chol-NBD and DOPC-Cy5 molecules for FRET; Figure S6: Fluorescence recovery with recognition of membrane cholesterol with quenched AuNP-βCD-Fl complexes; Figure S7: Fluorescence recovery after 2 h treatment of liposome particles with quenched AuNP-βCD-Fl complexes; Figure S8: Nanoparticle tracking analysis of DPPC/Chol liposomes before and after treatment with AuNP/mβCD; Figure S9: Fluorescence recovery with different concentrations of membrane cholesterol; Figure S10: PL response of AuNP/mβCD on DOPC/Chol liposomes for the cholesterol concentration expressed linear scales with x axis. Table S1: Calculation of recovered fluorescence with AuNPs-βCD-Fl interaction after recognition of membrane cholesterol; Video S1: Scattered light of DPPC/Chol liposome particles before and after treatment of quenched AuNP/mβCD.

## References

[1] Sau T.K., Rogach A.L., Jäckel F., Klar T.A., Feldmann J. (2010). Properties and applications of colloidal nonspherical noble metal nanoparticles. Adv Mater.

[2] Hu M., Chen J., Li Z.Y., Au L., Hartland G.V., Li X., Marquez M., Xia Y. (2006). Gold nanostructures: Engineering their plasmonic properties for biomedical applications. Chem. Soc. Rev..

[3] Zeng S., Yong K.-T., Roy I., Dinh X.-Q., Yu X., Luan F. (2011). A review on functionalized gold nanoparticles for biosensing applications. Plasmonics.

[4] Mulvaney P. (1996). Surface plasmon spectroscopy of nanosized metal particles. Langmuir.

[5] Shukla R., Bansal V., Chaudhary M., Basu A., Bhonde R.R., Sastry M. (2005). Biocompatibility of gold nanoparticles and their endocytotic fate inside the cellular compartment: A microscopic overview. Langmuir ACS J. Surf. Colloids.

[6] Li X., Huang J., Wu X., Piao X., Lv D., Zhang G., Ahn D.J., Cui C. (2023). Obviously Enhanced Fluorescent Signal of Core-Shell Nanostructures through Simultaneous Regulation of Spectral Overlap and Shell Thickness for Imaging and Photothermal Therapy of Ovarian Cancer Cells. Part. Part. Syst. Charact..

[7] Kus-Liśkiewicz M., Fickers P., Ben Tahar I. (2021). Biocompatibility and Cytotoxicity of Gold Nanoparticles: Recent Advances in Methodologies and Regulations. Int. J. Mol. Sci..

[8] Templeton A.C., Wuelfing W.P., Murray R.W. (2000). Monolayer-protected cluster molecules. Acc. Chem. Res..

[9] Chen Y., Xianyu Y., Jiang X. (2017). Surface modification of gold nanoparticles with small molecules for biochemical analysis. Acc. Chem. Res..

[10] Bozich J.S., Lohse S.E., Torelli M.D., Murphy C.J., Hamers R.J., Klaper R.D. (2014). Surface chemistry, charge and ligand type impact the toxicity of gold nanoparticles to Daphnia magna. Environ. Sci. Nano.

[11] Zhang J., Mou L., Jiang X. (2020). Surface chemistry of gold nanoparticles for health-related applications. Chem. Sci..

[12] Mirkin C.A., Letsinger R.L., Mucic R.C., Storhoff J.J. (1996). A DNA-based method for rationally assembling nanoparticles into macroscopic materials. Nature.

[13] Elghanian R., Storhoff J.J., Mucic R.C., Letsinger R.L., Mirkin C.A. (1997). Selective colorimetric detection of polynucleotides based on the distance-dependent optical properties of gold nanoparticles. Science.

[14] Pengo P., Broxterman Q.B., Kaptein B., Pasquato L., Scrimin P. (2003). Synthesis of a stable helical peptide and grafting on gold nanoparticles. Langmuir.

[15] Schneider B.H., Dickinson E.L., Vach M.D., Hoijer J.V., Howard L.V. (2000). Highly sensitive optical chip immunoassays in human serum. Biosens. Bioelectron..

[16] Thanh N.T., Rosenzweig Z. (2002). Development of an aggregation-based immunoassay for anti-protein A using gold nanoparticles. Anal. Chem..

[17] Bhattacharya S., Srivastava A. (2003). Synthesis and characterization of novel cationic lipid and cholesterol-coated gold nanoparticles and their interactions with dipalmitoylphosphatidylcholine membranes. Langmuir.

[18] Gole A., Dash C., Soman C., Sainkar S.R., Rao M., Sastry M. (2001). On the preparation, characterization, and enzymatic activity of fungal protease-gold colloid bioconjugates. Bioconjugate Chem..

[19] Yun C.S., Javier A., Jennings T., Fisher M., Hira S., Peterson S., Hopkins B., Reich N.O., Strouse G.F. (2005). Nanometal surface energy transfer in optical rulers, breaking the FRET barrier. J. Am. Chem. Soc..

[20] Chen C., Hildebrandt N. (2020). Resonance energy transfer to gold nanoparticles: NSET defeats FRET. TrAC Trends Anal. Chem..

[21] Sen T., Patra A. (2012). Recent advances in energy transfer processes in gold-nanoparticle-based assemblies. J. Phys. Chem. C.

[22] Lakowicz J.R. (2006). Principles of Fluorescence Spectroscopy.

[23] Förster T. (1948). Zwischenmolekulare energiewanderung und fluoreszenz. Ann. Der Phys..

[24] Di Nunzio M.R., Douhal A. (2023). Robust Inclusion Complex of Topotecan Comprised within a Rhodamine-Labeled β-Cyclodextrin: Competing Proton and Energy Transfer Processes. Pharmaceutics.

[25] Zhao X., Gao J., Song Y., Zhang J., Han Q. (2022). Determination of Fumonisin B1 by Aptamer-Based Fluorescence Resonance Energy Transfer. Sensors.

[26] Chen G., Jiang M. (2011). Cyclodextrin-based inclusion complexation bridging supramolecular chemistry and macromolecular self-assembly. Chem. Soc. Rev..

[27] Schmidt B.V.K.J., Barner-Kowollik C. (2017). Dynamic Macromolecular Material Design-The Versatility of Cyclodextrin-Based Host-Guest Chemistry. Angew. Chem. (Int. Ed. Engl.).

[28] Szente L., Fenyvesi É. (2017). Cyclodextrin-lipid complexes: Cavity size matters. Struct. Chem..

[29] Huang Z., London E. (2013). Effect of cyclodextrin and membrane lipid structure upon cyclodextrin-lipid interaction. Langmuir ACS J. Surf. Colloids.

[30] Yadav H.M., Park J.-D., Kang H.-C., Lee J.-J. (2021). Recent development in nanomaterial-based electrochemical sensors for cholesterol detection. Chemosensors.

[31] Mondal A., Jana N.R. (2012). Fluorescent detection of cholesterol using β-cyclodextrin functionalized graphene. Chem. Commun. (Camb. Engl.).

[32] Peng J., Wang Y., Wang J., Zhou X., Liu Z. (2011). A new biosensor for glucose determination in serum based on up-converting fluorescence resonance energy transfer. Biosens. Bioelectron..

[33] Flamigni L. (1993). Inclusion of fluorescein and halogenated derivatives in. alpha-,. beta-, and gamma-cyclodextrins: A steady-state and picosecond time-resolved study. J. Phys. Chem..

[34] Breslow R., Zhang B. (1996). Cholesterol recognition and binding by cyclodextrin dimers. J. Am. Chem. Soc..

[35] Goh D.Y., Ahn D.J. (1997). Carbonate crystal growth controlled by interfacial interactions of artificial cell membranes. Biotechnol. Bioprocess Eng..

[36] Won T.K., Roh J., Ahn D.J. (2022). Fabrication of long-lasting multilayers of diacetylene@ silica nanoparticles patterned on solids for sensory figures. J. Ind. Eng. Chem..

[37] Seo J.S., Liu H., Cho Y.H., Jung W.H., Kim S., Ahn D.J. (2023). Triple-Peak Photoluminescence of DNA-Hybrid Alq3 Crystals Emitting a Depressed Single Peak upon Bio-Recognition. ACS Appl. Mater. Interfaces.

[38] Shin S., Cho Y.H., Park J.H., Ahn D.J. (2022). Light-emitting crystals of aptamer-hybrid organic semiconductor signaling on human cells expressing EpCAM. J. Ind. Eng. Chem..

[39] Frens G. (1973). Controlled nucleation for the regulation of the particle size in monodisperse gold suspensions. Nat. Phys. Sci..

[40] Stetefeld J., McKenna S.A., Patel T.R. (2016). Dynamic light scattering: A practical guide and applications in biomedical sciences. Biophys. Rev..

[41] Lim J., Yeap S.P., Che H.X., Low S.C. (2013). Characterization of magnetic nanoparticle by dynamic light scattering. Nanoscale Res. Lett..

