# Supplementary material for: Energy Transfer-Based Recognition of Membrane Cholesterol by Controlling Intradistance of Linker
Source: Sensors (Basel). 2024 Apr 5;24(7):2315. doi: 10.3390/s24072315 (PMC11014005; doi:10.3390/s24072315)
Supplement: Supplementary file 1 [file sensors-24-02315-s001.zip › sensors-2928842-supplementary.pdf]

Supplementary Information

Energy Transfer-Based Recognition of Membrane Cholesterol by Controlling Intradistance of Linker

Yong Ho Cho [1], Tae Kyung Won [1] and Dong June Ahn [1,2,*]

[1] Department of Chemical and Biological Engineering, Korea University, Seoul 02841, Republic of Korea; cyh9312@korea.ac.kr (Y.H.C.); callys@korea.ac.kr (T.K.W.)
[2] KU-KIST Graduate School of Converging Science and Technology, Korea University, Seoul 02841, Republic of Korea
* Correspondence: ahn@korea.ac.kr; Tel.: +82-02-3290-3301

Table of contents

Supplementary Figures 1–10
Supplementary Table 1
Supplementary Video 1

Supplementary Figures

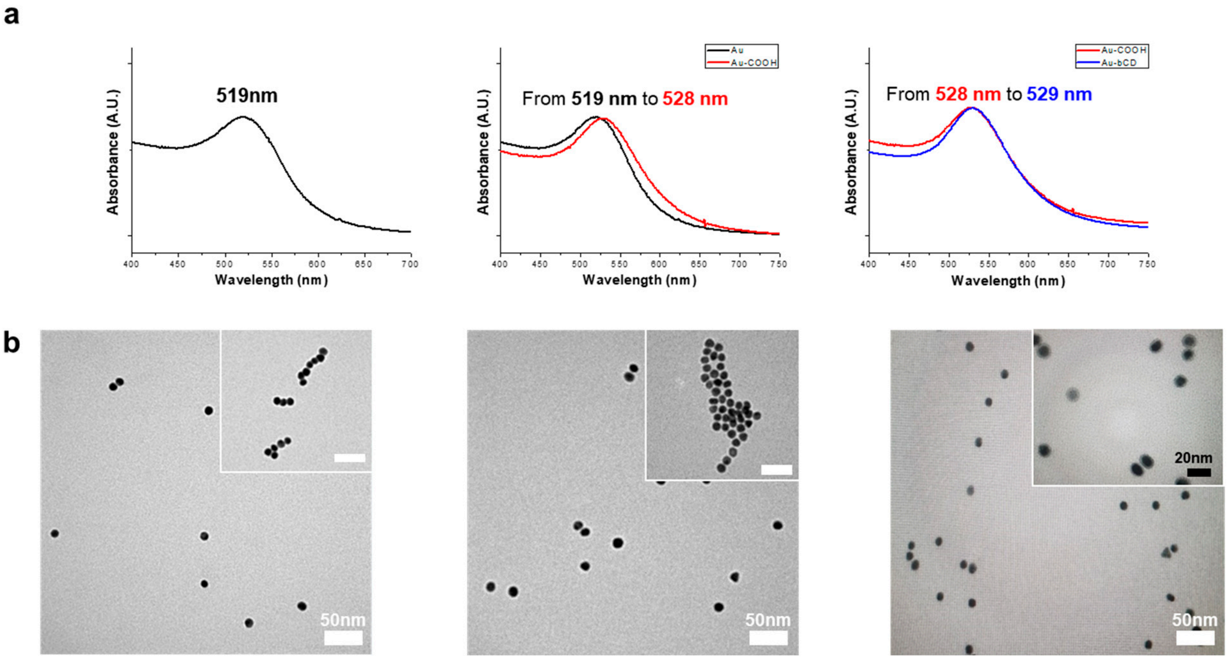

Figure S1. Analysis of visible spectroscopy and transmittance electron microscopy (TEM) images in the process of AuNP/C12/βCD fabrication. (a) Absorption spectra and (b) TEM images of AuNP/Citrate, AuNP/C12, and AuNP/C12/βCD particles.

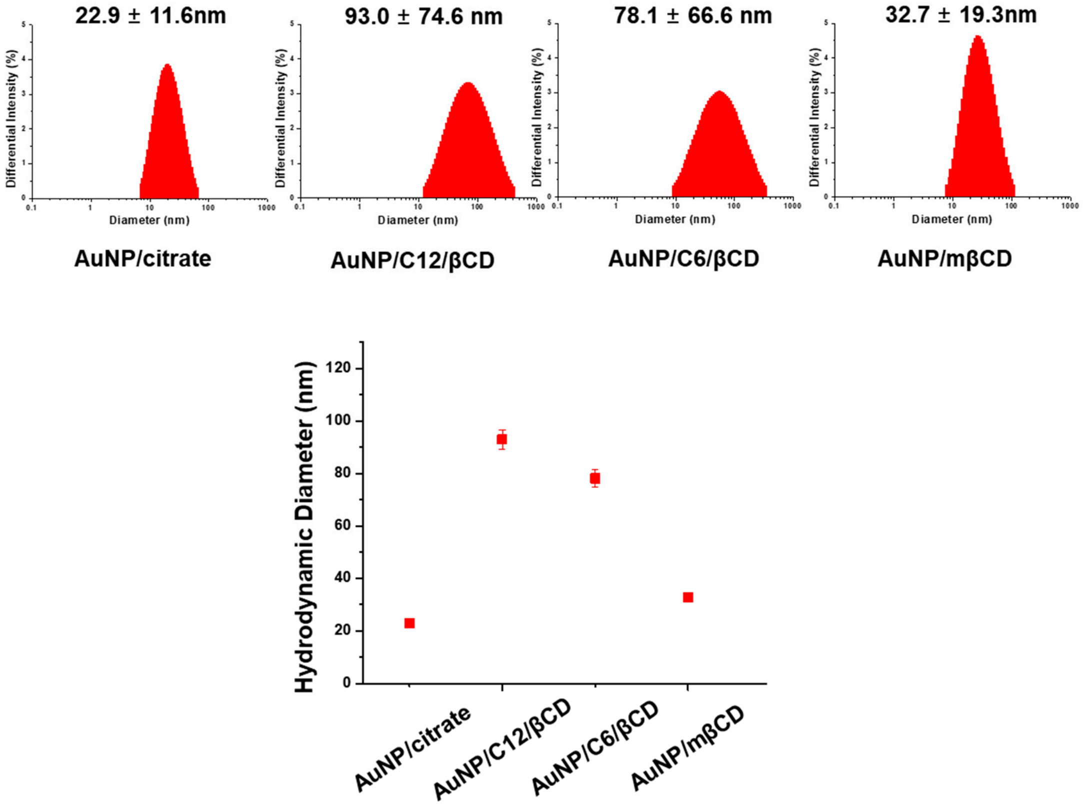

Figure S2. Analysis of dynamic light scattering with AuNP-βCD particles. Hydrodynamic diameters of AuNP/citrate, AuNP/C12/βCD, AuNP/C6/βCD, and AuNP/mβCD.

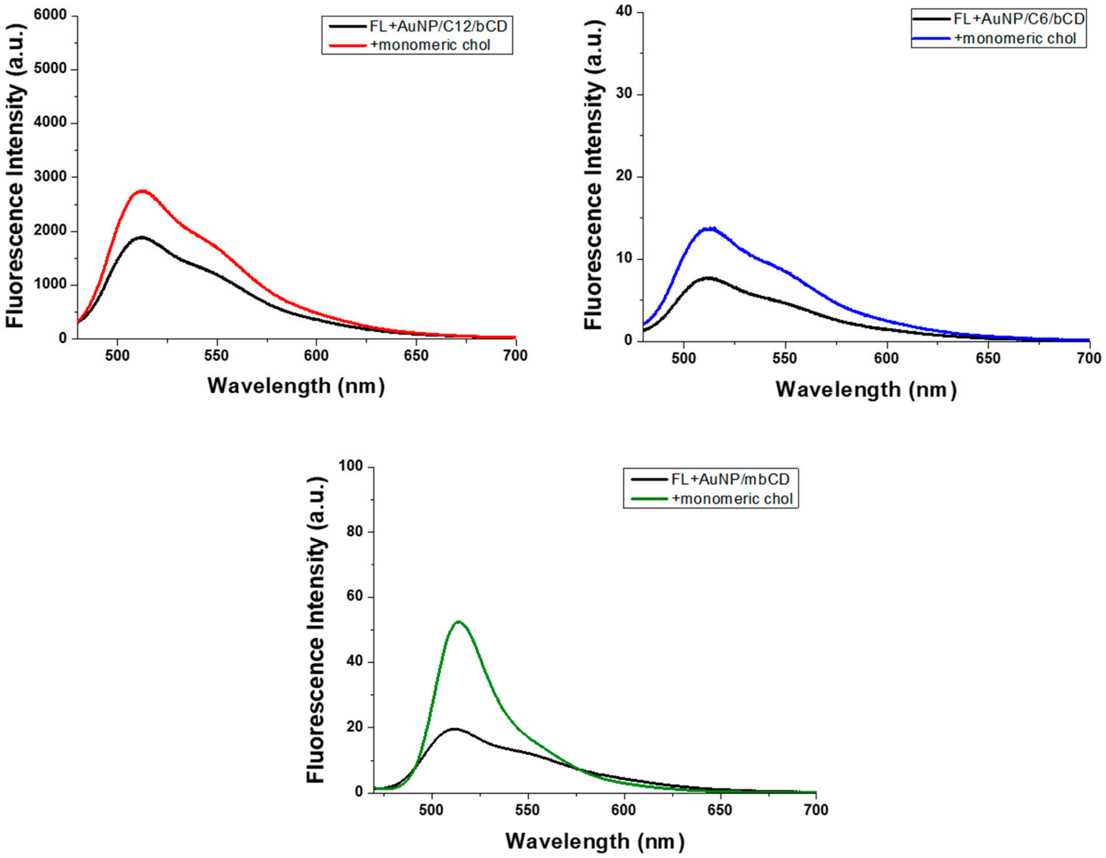

Figure S3. Photoluminescence spectra for recognition of monomeric cholesterol. Fluorescence recovery of AuNP-βCD-Fl particles before and after treatment with monomeric cholesterol.

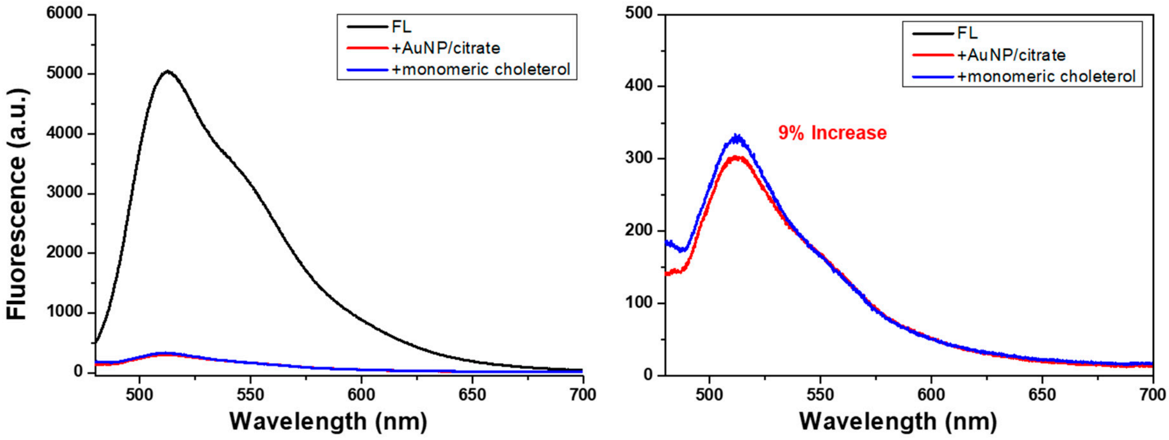

Figure S4. Photoluminescence spectra of fluorescence quenching and turn-on recovery process with AuNP/Citrate. PL intensity of fluorescence of Fl quenched with AuNP/Citrate and recovered without specific recognition of monomeric cholesterol.

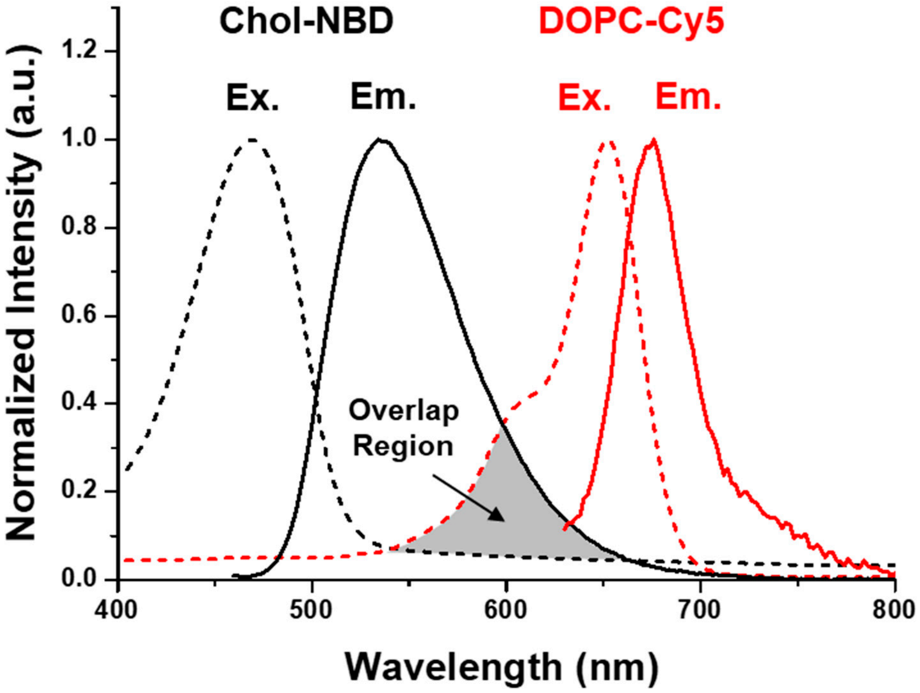

Figure S5. Optical properties of Chol-NBD and DOPC-Cy5 molecules for FRET. Excitation and emission spectra of Chol-NBD (donor) and DOPC-Cy5 (acceptor) molecules for FRET. There was an overlap region between the emission band of donor and excitation band of acceptor.

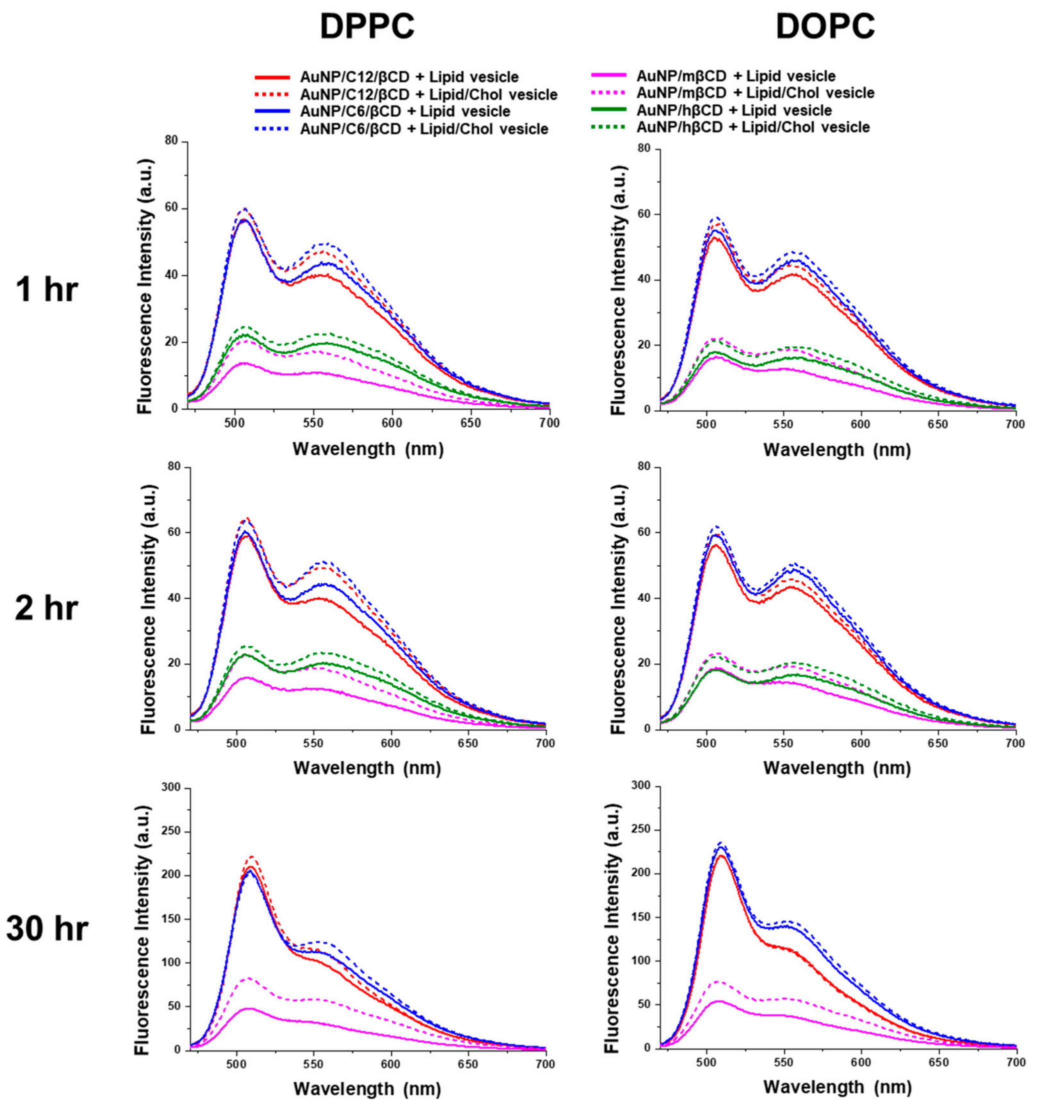

Figure S6. Fluorescence recovery with recognition of membrane cholesterol with quenched AuNP-βCD-Fl complexes. Photoluminescence spectra before and after treatment of four types of liposome particles (DPPC, DPPC/Chol, DOPC, and DOPC/Chol) for 1, 2, and 30 h with quenched AuNP-βCD-Fl complexes.

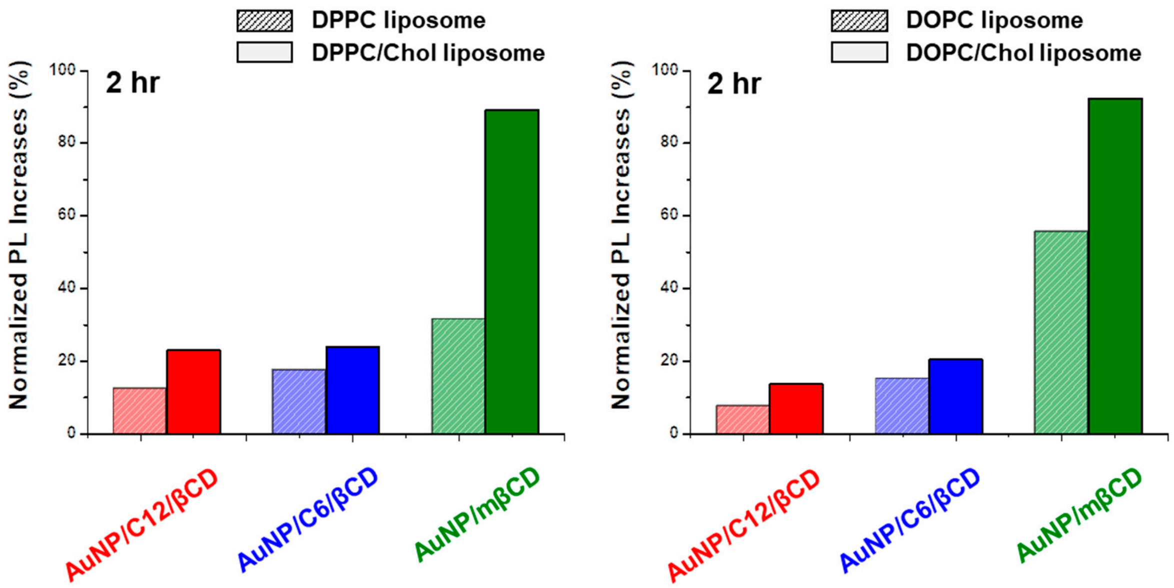

Figure S7. Fluorescence recovery after 2 h treatment of liposome particles with quenched AuNP-βCD-Fl complexes. The normalized PL increases of quenched AuNP/βCD particles after 2 h treatment of liposome particles.

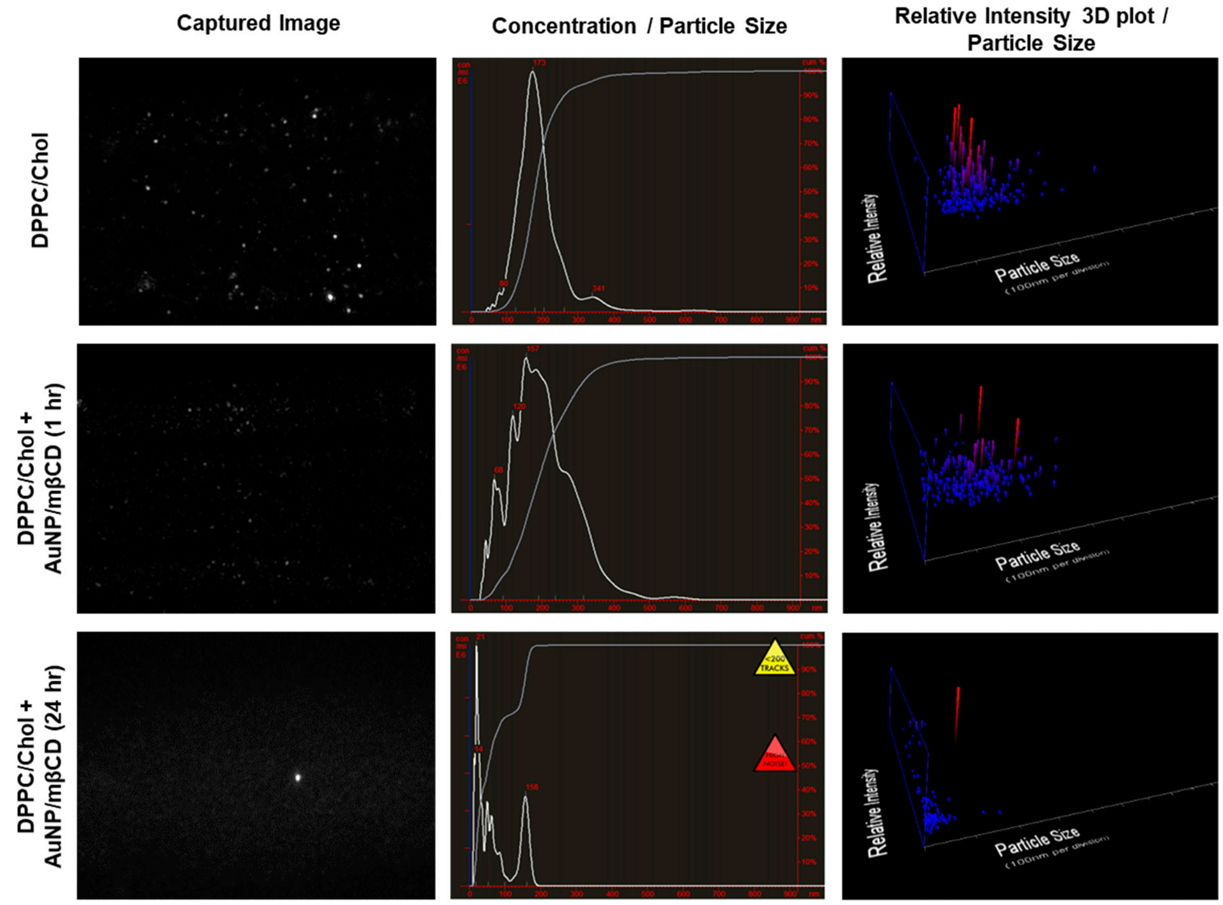

Figure S8. Nanoparticle tracking analysis of DPPC/Chol liposomes before and after treatment with AuNP/mβCD. Captured images, size distribution, and 3D plot mapping of DPPC/Chol liposomes.

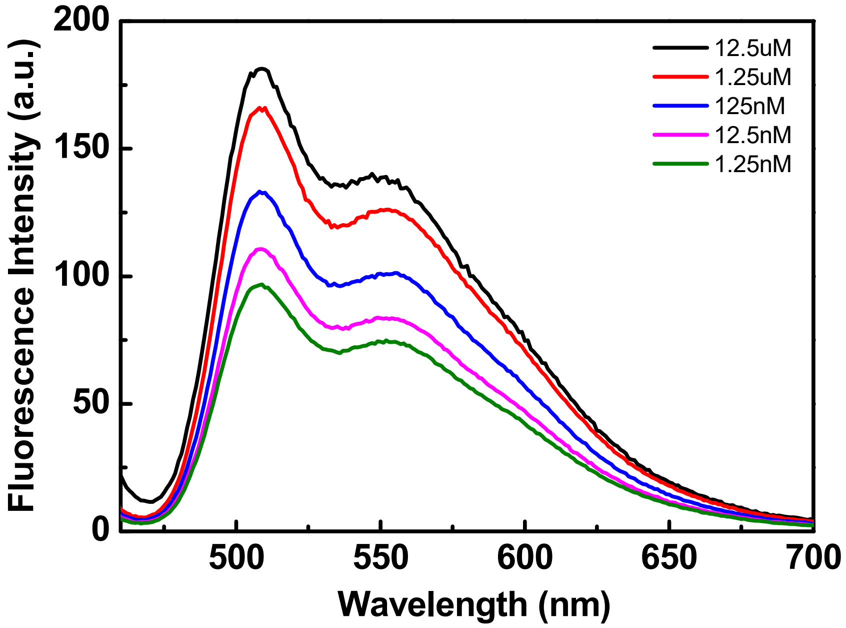

Figure S9. Fluorescence recovery with different concentrations of membrane cholesterol. Photoluminescence spectra with AuNP/mβCD-Fl complex after treatment with DOPC/Chol liposome.

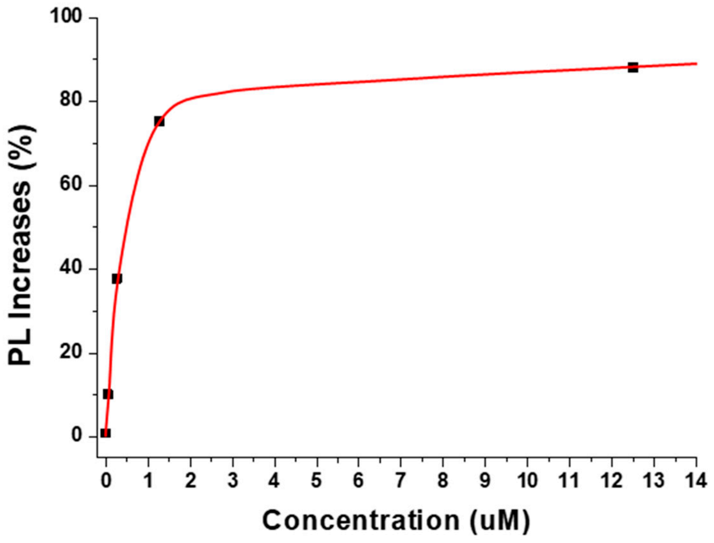

Figure S10. PL response of AuNP/mβCD on DOPC/Chol liposomes for the cholesterol concentration expressed linear scales with x axis.

Supplementary Table

Table S1. Calculation of recovered fluorescence with AuNPs-βCD-Fl interaction after recognition of membrane cholesterol.

AuNP-βCD	Quenched PL intensity (I_o)	Normalized Pl Intensity for target molecules (I)					
		DPPC			DPPC/CHOL		
		1 h	2 h	30 h	1 h	2 h	30 h
AuNP/C12/βCD	52.43	56.82	58.95	210.30	60.14	64.56	221.84
PL increasing(%)	-	8.4	12.4	301.1	14.7	23.1	323.1
AuNP/C6/βCD	51.44	56.49	60.49	207.64	60.09	63.74	202.25
PL increasing(%)	-	9.8	17.6	303.7	16.8	23.9	293.2
AuNP/mβCD	12.12	13.79	15.95	48.07	20.55	22.93	82.93
PL increasing(%)	-	13.8	31.6	296.6	69.6	89.2	584.3
		DOPC			DOPC/CHOL		
		1 h	2 h	30 h	1 h	2 h	30 h
AuNP/C12/βCD	52.43	52.98	56.42	220.98	57.11	59.62	210.44
PL increasing(%)	-	1.0	7.6	321.4	8.9	13.7	301.3
AuNP/C6/βCD	51.44	55.20	59.28	230.12	59.06	61.99	235.83
PL increasing(%)	-	7.3	15.2	347.3	14.8	20.5	358.4
AuNP/mβCD	12.12	16.59	18.86	54.29	22.39	23.32	77.06
PL increasing(%)	-	36.9	55.6	348.0	84.7	92.4	535.9

Supplementary Video

Video S1. Scattered light of DPPC/Chol liposome particles before and after treatment (1 and 30 hr) of quenched AuNP/mβCD.
